# Effects of replacing soybean meal with powdered or pelleted black soldier fly larvae on nutrient digestibility and rumen fermentation in Thai native beef cattle

**DOI:** 10.5713/ab.25.0245

**Published:** 2025-07-11

**Authors:** Nittaya Phowang, Chanon Suntara, Anusorn Cherdthong

**Affiliations:** 1 Tropical Feed Resources Research and Development Center (TROFREC), Department of Animal Science, Faculty of Agriculture, Khon Kaen University, Khon Kaen, Thailand

**Keywords:** Feed Processing, Insect Protein, Rumen Dynamic, Sustainability

## Abstract

**Objective:**

This study evaluated the effects of replacing soybean meal (SBM) with black soldier fly larvae (BSFL) in powdered and pelleted forms on feed intake, rumen fermentation, and nutrient digestibility in Thai native beef cattle.

**Methods:**

Four male Thai native beef cattle (3–3.5 years old; 370±20.0 kg body weight) were assigned to a 4×4 Latin square design to evaluate the effects of replacing SBM with BSFL in the concentrate portion of the diet. The dietary treatments were: T1, control diet with SBM as the sole protein source; T2, 50% of SBM replaced with powdered BSFL; T3, 25% of SBM replaced with pelleted BSFL; and T4, 75% of SBM replaced with pelleted BSFL.

**Results:**

Replacing SBM with BSFL had no significant effect on dry matter (DM) intake, ruminal pH, ammonia-nitrogen, or volatile fatty acid concentrations (p>0.05). Neutral detergent fiber intake was significantly higher in cattle fed the diet containing 75% pelleted BSFL compared to those fed 25% pelleted BSFL (p<0.01). Crude protein digestibility improved overall in BSFL-supplemented groups compared to the control (p<0.01). The highest DM digestibility was observed at 25% pelleted BSFL, significantly higher than at 75% inclusion (p<0.05). At 4 hours post-feeding, blood urea nitrogen concentration was significantly lower in cattle fed the 75% pelleted BSFL diet compared to those fed 25% pelleted BSFL (p<0.05). Protozoal populations, total volatile fatty acid concentrations, and the volatile fatty acid profile did not differ significantly among treatments (p>0.05).

**Conclusion:**

Replacing SBM with BSFL, particularly at 25% in pelleted form, improves nutrient digestibility without impairing rumen fermentation, supporting its potential as a functional protein source in ruminant diets.

## INTRODUCTION

Global population growth and economic development are expected to continue rising rapidly, increasing the global demand for meat and milk [1]. This increasing demand has placed pressure on feed resources, especially protein sources, leading to higher feed costs and ultimately driving up livestock production expenses [2]. As a result, identifying alternative protein sources that are economically viable, nutritionally adequate, and environmentally sustainable is a growing priority for the livestock industry. In this context, black soldier fly larvae (BSFL) have gained increasing attention as a promising alternative to conventional protein sources in animal feeds.

BSFL are rich in crude protein (CP), fat, essential amino acids, and minerals [3], with CP content ranging from 46.6% to 59.9% of DM—comparable to soybean meal (SBM) and fishmeal [3–5]. Moreover, BSFL contain unique bioactive compounds, particularly antimicrobial peptides such as defensin, lysozyme, attacin, and knottin-like peptides (44%, 18%, 7%, and 5%, respectively) [6], which are not typically found in plant-based protein sources like SBM. These functional components may support gut health and enhance microbial activity in the rumen [5,6].

Recent studies have begun exploring the potential of BSFL in ruminant diets. An *in vitro* study by Kahraman et al [7] showed that replacing SBM with BSFL at 20% and 40% in total mixed rations increased volatile fatty acid (VFA) concentrations and ammonia-nitrogen (NH_3_–N), along with improved digestibility. Similarly, Jayanegara et al [8] reported that replacing 40% of SBM with BSFL elevated VFA production while reducing NH_3_–N and *in vitro* methane (CH_4_) emissions, suggesting enhanced microbial protein synthesis and improved nitrogen utilization. These findings indicate that BSFL inclusion may benefit rumen fermentation and contribute to environmental sustainability by reducing CH_4_ production [8]. However, most studies are based on *in vitro* models and do not account for how substrate-dependent variations in BSFL composition may affect *in vivo* digestibility, nutrient use, or rumen fermentation [5].

Although prior studies have shown promise, most research on BSFL has been conducted *in vitro* or in monogastric models, limiting its applicability to ruminant feeding under *in vivo* conditions [3,5,8]. In particular, there is limited understanding of how the form (powdered *vs*. pelleted) influences digestibility and rumen fermentation in ruminants. Furthermore, indicators like blood metabolites and protozoal populations, which offer deeper insights into metabolic and microbial dynamics, have received little attention. It also remains unclear whether the processing form (e.g., pelleting) interacts with inclusion rate to affect nutrient utilization efficiency. This study seeks to fill those gaps by providing *in vivo* evidence on the nutritional and physiological outcomes of feeding BSFL-based diets to beef cattle.

Therefore, the objective of this study was to evaluate the effects of partially replacing SBM with BSFL in powdered and pelleted forms on dry matter intake (DMI), rumen fermentation parameters, nutrient digestibility, blood urea nitrogen (BUN), and protozoal populations in Thai native beef cattle. This research aims to clarify whether BSFL can serve as a functional and sustainable protein alternative in ruminant diets.

## MATERIALS AND METHODS

### Animals and dietary treatments

All experimental procedures were approved by the Animal Ethics Committee of Khon Kaen University (Approval No. IACUC-KKU-100/66). Four male Thai native beef cattle, aged 3–3.5 years and with an average initial body weight of 370± 20.00 kg, were randomly assigned to a 4×4 Latin square design. The four dietary treatments were selected based on results from our previous preliminary *in vitro* study and included: T1, a control group in which SBM served as the sole protein source; T2, a diet replacing 50% of SBM with powdered BSFL; T3, a diet replacing 25% of SBM with pelleted BSFL; and T4, a diet replacing 75% of SBM with pelleted BSFL. BSFL used in the study was provided by BSFLY, Ban Phaeng District, Nakhon Phanom Province, Thailand. The pelleting process involved mixing powdered BSFL with tapioca starch at a 10:1 ratio to promote gelatinization, adjusting moisture to 50%, and then passing the mixture through a 4 mm die pelletizer at 60°C–65°C. The resulting pellets were long, cylindrical, and sun-dried for approximately 72 hours, following the procedures of Sommai et al [9] and Seankamsorn and Cherdthong [10]. All animals were housed in individual pens with access to clean drinking water and mineral blocks at all times. Concentrate was offered at 1% of body weight daily, while rice straw was supplied ad libitum at 07:00 and 16:00 h. The experiment consisted of four 21-day periods, each comprising 14 days for dietary adaptation and 7 days for data and sample collection. During the final 7 days of each period, the animals were transferred to metabolism cages for total fecal collection to evaluate nutrient digestibility. The proportions of ingredients and their chemical composition for the concentrate and rice straw are summarized in Table 1.

### Data and sample collection

Feed intake was recorded daily by weighing the offered and refused portions of both roughage and concentrate in the morning prior to feeding. During the last 7 days of each experimental period, daily samples of concentrate, roughage, orts, and feces were collected. Since the animals were housed in metabolism cages for digestibility evaluation, approximately 5% of the total fresh fecal output was subsampled daily and separated into two portions: one portion was used for immediate DM analysis, while the other was stored in a refrigerator and later pooled by individual animal at the end of each period for chemical analysis. All samples were dried at 60°C and ground to pass through a 1 mm sieve using a Cyclotech Mill (Tecator, Hoganas). Chemical composition of the diets was analyzed for DM, ash, ether extract, and CP following the procedures of AOAC [11], while neutral detergent fiber (NDF) and acid detergent fiber were determined according to the method of Van Soest et al [12]. At the end of each period, rumen fluid and blood samples were collected at two time points: 0 h (before morning feeding) and 4 h post-feeding. Rumen fluid (100 mL) was obtained via stomach tubing using a vacuum pump and immediately divided into three subsamples. The first portion was immediately used to measure pH with a portable pH meter (HANNA HI 83141, HANNA Instruments). The second portion (approximately 40 mL) was preserved by adding 1 mL of 1 M H_2_SO_4_ per 10 mL of rumen fluid to inhibit microbial activity, then centrifuged at 3,000 rpm for 15 minutes. The clear supernatant (20–35 mL) was stored at −20°C for subsequent analysis of ammonia-nitrogen (NH_3_–N). The third portion consisted of 1 mL of uncentrifuged rumen fluid mixed with 9 mL of formalin and stored at 4°C for protozoal enumeration using the direct count method described by Galyean [13]. Additionally, VFAs, including acetic acid (C2), propionic acid (C3), and butyric acid (C4), were analyzed using gas chromatography (Nexis GC-2030; Shimadzu), following the protocol of Khota et al [14]. Blood samples (5 mL) were collected from the jugular vein into EDTA-containing tubes and centrifuged at 3,000 rpm; the resulting plasma fraction was separated and analyzed for BUN according to standard procedures [15].

### Statistical data analysis

All data were analyzed using analysis of variance (ANOVA) based on a 4×4 Latin square design using the PROC GLM procedure in SAS software [16]. The statistical model included diet as a fixed effect, animal as a random effect, and period as a fixed effect to account for variation across individual animals and experimental periods, which is particularly important given the small sample size (n = 4). Treatment effects were evaluated using orthogonal contrasts specifically designed to address the study’s main objectives: (1) comparison between the control group (T1) and all BSFL-supplemented groups (T2, T3, T4), and (2) comparison between different inclusion levels of pelleted BSFL (T3 vs. T4). Post-hoc pairwise comparisons such as Tukey’s HSD test were not performed, as the orthogonal contrasts sufficiently addressed the predefined hypotheses and minimized the risk of Type I error. Differences were considered statistically significant at p<0.05. The model used was:


(1) $$Y_{ijk}=\mu +\rho_{i}+\gamma_{j}+\tau_{k}+{ɛ}_{ijk}$$

Where: *Y_ijk_*: observation for diet *i*, animal *j*, in period *k*; μ: overall mean; *ρ_i_*: fixed effect of dietary treatment (*i* = 1 to 4); *γ_j_*: random effect of animal (*j* = 1 to 4); *τ_k_*: fixed effect of period (*k* = 1 to 4); *ɛ_ijk_*: residual error term.

## RESULTS

### Feed intake, nutrient intake, and nutrient digestibility

The effects of replacing SBM with BSFL on feed intake and nutrient utilization in Thai native beef cattle are presented in Table 2. Dietary replacement of SBM with BSFL did not significantly affect total DMI or intake of individual feed components (p>0.05). Total DM intake ranged from 8.86 to 9.38 kg/day, with rice straw and concentrate intake ranging from 4.43 to 4.80 kg/day and 4.35 to 4.65 kg/day, respectively. Similarly, there were no significant differences in OM and CP intake among treatments (p>0.05), with OM intake ranging from 7.68 to 8.18 kg/day and CP intake from 0.78 to 0.82 kg/day. A significant difference was observed in NDF intake (p<0.01), with cattle fed 75% pelleted BSFL showing higher intake compared to those fed 25% pelleted BSFL.

Table 3 presents the effects of BSFL inclusion on nutrient digestibility. Replacing SBM with BSFL significantly increased DM digestibility (p<0.05). Among the pelleted BSFL groups, the 25% replacement group had higher DM digestibility than the 75% replacement group. In addition, CP digestibility was significantly improved (p<0.05) in each of the BSFL-supplemented groups compared to the control, suggesting enhanced protein utilization with BSFL inclusion.

### Ruminal fermentation, blood metabolite, and protozoal population

The effects of SBM with BSFL on ruminal fermentation, blood metabolites, and protozoal population in Thai native beef cattle are presented in Table 4. The replacement of SBM with BSFL in the concentrate diet did not significantly affect ruminal pH or NH_3_–N concentrations (p>0.05). Ruminal pH averaged between 6.81 and 6.97, and NH_3_–N concentrations ranged from 12.3 to 13.3 mg/dL among all treatments. Blood urea-nitrogen concentrations were significantly affected (p<0.05) by the BSFL inclusion level in the pelleted treatments, with cattle fed 75% pelleted BSFL showing lower BUN concentrations than those fed 25% pelleted BSFL.

Protozoal populations ranged from 1.25 to 1.56 log_10_ cell/mL at 0 h pre-feeding and from 1.41 to 1.88 log_10_ cell/mL at 4 h post-feeding. No significant differences were observed among treatments at either time point (p>0.05).

### Total volatile fatty acids and volatile fatty acid profile

The effects of replacing SBM with BSFL on total VFA (TVFA) and VFA profile (VFAP) in Thai native beef cattle are presented in Table 5. Inclusion of BSFL in the concentrate diet did not significantly affect TVFA concentration or individual VFAs (C2, C3, and C4), nor the C2-to-C3 ratio, at either 0 or 4 hours post-feeding (p>0.05). The mean TVFA concentrations ranged from 100.3 to 104.9 mmol/L. The proportion of C2 ranged from 61.0% to 65.9% of TVFA, C3 from 20.9% to 25.3% of TVFA, and C4 from 12.7% to 14.6% of TVFA. The C2:C3 ratio ranged from 2.40 to 3.10.

## DISCUSSION

### Feed intake, nutrient intake, and nutrient digestibility

The results of this study indicate that the total DMI of Thai native beef cattle fed rice straw-based diets was within the expected range based on their body weight, suggesting that replacing SBM with BSFL did not negatively impact feed palatability or intake. These findings are consistent with earlier research showing that alternative protein sources may not reduce intake if nutrient density is preserved [10].

Notably, both the physical form of BSFL (pelleted) and its inclusion level appear to influence nutrient utilization efficiency through multiple interacting mechanisms. BSFL provide high-quality protein with a favorable amino acid profile, which supports microbial growth and enzymatic activity in the rumen, thereby enhancing nutrient digestibility [3,4,17]. Pelleting enhances feed uniformity and bulk density and induces partial starch gelatinization, which may facilitate microbial access, thereby improving fermentation efficiency. Although high inclusion levels of BSFL (e.g., 75%) are often associated with reduced rumen fermentation due to their higher fat or chitin content, this study found no significant differences in fermentation parameters between the pelleted treatments. This suggests that the observed improvement in nutrient digestibility is likely attributed to the moderate inclusion level (25%) and the enhanced physical properties from pelleting, rather than a dose-dependent response to BSFL. These findings align with the report of Phesatcha et al [18], who found improved protein digestibility when SBM was partially replaced with cricket meal in beef cattle diets. Therefore, the enhanced digestibility observed in the present study likely reflects a balanced interaction between inclusion rate and feed physical structure, which together promote favorable rumen fermentation conditions.

### Ruminal fermentation, blood metabolite, and protozoal population

The ruminal pH observed in this study remained within the optimal physiological range of 6.5 to 7.0, which supports efficient microbial fermentation of dietary fiber and protein [19]. This finding indicates that partial replacement of SBM with BSFL did not negatively affect the ruminal environment. Similarly, ruminal NH_3_–N concentrations ranged from 12.3 to 13.3 mg/dL, which is considered adequate for optimal microbial protein synthesis and fiber digestion. According to Perdok and Leng [20], the optimal NH_3_–N concentration in the rumen lies between 5.0 and 25.0 mg/dL. Other studies suggest that a range of 15–30 mg/dL is ideal for maximizing fermentation and microbial growth [20,21]. Therefore, the NH_3_–N levels observed in this experiment indicate stable nitrogen metabolism and microbial activity.

At 4 hours post-feeding, BUN concentrations were significantly lower in cattle fed the 75% pelleted BSFL diet compared to those receiving the 25% inclusion level, indicating reduced circulating nitrogen at this time point. Although lower BUN levels are generally interpreted as a sign of improved nitrogen retention, this was not accompanied by increased digestibility in the 75% BSFL group. In fact, both DM and CP digestibility declined, suggesting that excessive BSFL inclusion may have impaired nitrogen utilization. This could be due to elevated dietary fat or chitin levels associated with higher BSFL inclusion, both of which are known to negatively impact rumen microbial function and nutrient absorption [10,15]. Elevated fat can inhibit fiber-degrading bacteria, while chitin, being a complex and poorly digestible polysaccharide, may interfere with microbial protein synthesis [8]. In contrast, the higher BUN concentrations observed in the 25% pelleted BSFL group may reflect a more favorable nitrogen-energy synchronization that supports efficient microbial protein synthesis [21]. This interpretation is supported by the superior nutrient digestibility observed in this group (Table 3), indicating that moderate inclusion of BSFL promotes better nutrient availability and microbial efficiency [22]. Thus, despite the lower BUN in the 75% group, the overall physiological response favors moderate BSFL inclusion for optimizing rumen nitrogen metabolism and feed utilization.

### Total volatile fatty acids and volatile fatty acid profile

VFAs are the primary end-products of ruminal fermentation and contribute significantly to the energy supply of ruminants, accounting for approximately 40% to 70% of the digestible energy in the diet [23]. The major VFAs—C2, C3, and C4—are typically produced in molar proportions of 60%–70% acetate, 15%–20% propionate, and 10%–15% butyrate, although these values can vary depending on the composition of the diet [24,25]. Diets high in fiber tend to favor acetate production, whereas high-starch diets promote increased propionate levels. The balance among these VFAs plays a critical role in maintaining rumen function and meeting the host animal’s energy demands [26,27].

In the present study, the inclusion of BSFL—either in powdered or pelleted form—did not significantly alter total VFA concentrations or the VFA profile in Thai native beef cattle. The VFA levels remained within the typical physiological range reported in the literature [24], indicating stable ruminal fermentation. These findings are consistent with those of Fukuda et al [28], who reported no changes in VFA concentrations when SBM was replaced with BSFL in beef cattle diets.

Importantly, this study represents the first *in vivo* investigation evaluating the effects of pelleted BSFL as a protein source in ruminant diets. The results suggest that BSFL, particularly in pelleted form, can serve as a viable replacement for SBM without adversely affecting ruminal fermentation characteristics. However, given the limited data currently available on BSFL use in ruminants, especially in pelleted form, further *in vivo* research is warranted to validate these findings across different production systems and feeding conditions.

Beyond nutritional benefits, BSFL may offer economic advantages by utilizing low-cost organic waste as a substrate, potentially lowering feed costs [2,4]. Its local production can reduce dependence on imported protein sources like SBM [8]. Although a detailed cost analysis was not conducted, the use of pelleted BSFL may enhance practicality and reduce handling costs. Future studies should explore the cost-effectiveness under commercial conditions.

## CONCLUSION

This study demonstrated that replacing SBM with BSFL, in either powdered or pelleted form, improved DM and CP digestibility in Thai native beef cattle. The 25% pelleted BSFL diet resulted in the highest DM digestibility and more favorable BUN levels compared to the 75% inclusion, indicating enhanced nitrogen utilization. Protozoal populations, total VFA concentrations, and the VFA profile were not significantly affected among treatments. These findings suggest that moderate levels of pelleted BSFL may serve as a functional and sustainable protein source in ruminant diets. Further research is warranted to evaluate its long-term effects on animal performance, carcass characteristics, and rumen microbial ecology.

## Figures and Tables

**Table 1:** The proportion of ingredients in concentrate and nutrient contents of concentrate, rice straw used in experiment

Item	T1	T2	T3	T4	Rice straw
Ingredients (% DM)					
Soybean meal	15.0	7.50	11.25	3.75	
Powdered BSFL	0.00	7.50	0.00	0.00	
Pelleted BSFL	0.00	0.00	3.75	11.25	
Rice bran	14.0	14.0	14.0	14.0	
Palm meal	14.0	15.0	12.0	14.0	
Cassava chip	54.0	53.0	56.0	54.0	
Salt	1.00	1.00	1.00	1.00	
Premix	1.00	1.00	1.00	1.00	
Urea	1.00	1.00	1.00	1.00	
Chemical composition					
DM (%)	95.7	95.6	95.3	94.9	95.4
Organic matter (%DM)	93.9	94.5	94.9	94.1	81.4
Ash (%DM)	6.10	5.50	5.10	5.10	18.6
Crude protein (%DM)	15.3	15.2	15.5	15.1	2.40
Ether extract (%DM)	4.23	6.16	4.54	5.66	2.10
Neutral detergent fiber (%DM)	28.3	35.9	26.9	36.6	72.0
Acid detergent fiber (%DM)	11.6	12.1	11.0	11.4	44.8

T1 = control group; T2 = replacement SBM with powdered BSFL 50%; T3 = replacement SBM with pelleted BSFL 25%; T4 = replacement SBM with pelleted BSFL 75%.

DM, dry matter; BSFL, black soldier fly larvae; SBM, soybean meal.

**Table 2:** Effect of replacement SBM with BSFL on feed intake and nutrient intake in Thai native cattle

Item	Treatment				SEM	Contrast^1)^	
	
	T1	T2	T3	T4		(^1)^	(2)
Feed intake (DM)							
Rice straw							
kg/day	4.56	4.43	4.80	4.75	0.21	0.84	0.94
% BW	1.06	1.17	1.06	1.12	0.09	0.63	0.64
g/kg BW^0.75^	48.4	51.6	52.6	51.1	4.94	0.57	0.83
Concentrate							
kg/day	4.65	4.43	4.35	4.64	0.20	0.47	0.34
% BW	1.08	1.15	1.06	1.11	0.04	0.65	0.41
g/kg BW^0.75^	49.3	51.1	47.6	50.1	1.93	0.89	0.38
Total intake							
kg/day	9.20	8.86	9.15	9.38	0.50	0.90	0.75
% BW	2.15	2.32	2.12	2.23	0.24	0.59	0.53
g/kg BW^0.75^	97.6	102.7	95.4	100.7	4.97	0.74	0.46
Nutrient intake (%DM)							
DM (%)	9.20	8.86	9.15	9.38	0.50	0.90	0.75
Organic matter	8.07	7.79	7.68	8.04	0.36	0.58	0.49
Crude protein	0.82	0.78	0.78	0.81	0.03	0.47	0.51
Neutral detergent fiber	4.60	4.70	4.32	5.11	0.25	0.70	<0.01
Acid detergent fiber	2.58	2.52	2.44	2.63	0.14	0.75	0.34

1) Contrast: (1) Compare the difference between T1 VS T2, T3, T4, (2) Compare the difference between T3 VS T4 (p<0.05, p<0.01), p<0.05 means statistically significant, p<0.01 means highly statistically significant, p>0.05 means no statistical difference; T1 = control group; T2 = replacement SBM with powdered BSFL 50%; T3 = replacement SBM with pelleted BSFL 25%; T4 = replacement SBM with pelleted BSFL 75%.

SBM, soybean meal; BSFL, black soldier fly larvae; SEM, standard error of the mean; DM, dry matter.

**Table 3:** Effect of replacement SBM with BSFL on nutrient digestibility in Thai native cattle

Item	Treatment				SEM	Contrast^1)^	
	
	T1	T2	T3	T4		(1)	(2)
Nutrient digestibility (%DM)							
DM (%)	70.2	73.2	77.6	71.7	1.03	<0.05	<0.05
Organic matter	73.7	75.6	78.1	74.3	2.12	0.40	0.30
Crude protein	62.1	68.7	71.1	66.6	1.13	<0.01	0.07
Neutral detergent fiber	62.1	60.1	68.4	60.5	2.67	0.17	0.69
Acid detergent fiber	49.2	49.4	40.3	46.8	4.57	0.52	0.40

1) Contrast: (1) Compare the difference between T1 VS T2, T3, T4, (2) Compare the difference between T3 VS T4 (p<0.05, p<0.01), p<0.05 means statistically significant, p<0.01 means highly statistically significant, p>0.05 means no statistical difference; T1 = control group; T2 = replacement SBM with powdered BSFL 50%; T3 = replacement SBM with pelleted BSFL 25%; T4 = replacement SBM with pelleted BSFL 75%.

SBM, soybean meal; BSFL, black soldier fly larvae; SEM, standard error of the mean; DM, dry matter.

**Table 4:** Effect of replacement SBM with BSFL on ruminal fermentation, blood metabolite, and protozoal population in Thai native cattle

Item	Treatment				SEM	Contrast^1)^	
	
	T1	T2	T3	T4		(^1)^	(2)
Ruminal pH							
0 h pre-feeding	6.95	7.11	6.94	7.08	0.09	0.42	0.29
4 h post-feeding	6.66	6.71	6.74	6.86	0.12	0.53	0.55
Ammonia-nitrogen (NH_3_-N; mg/dL)							
0 h pre-feeding	12.6	11.9	11.9	12.60	0.31	0.40	0.28
4 h post-feeding	14.0	14.7	12.6	13.30	0.70	0.68	0.46
Blood urea nitrogen (BUN; mg/dL)							
0 h pre-feeding	8.33	9.33	9.00	9.00	1.05	0.59	1.00
4 h post-feeding	11.5	10.5	11.5	8.5	0.35	0.08	<0.05
Protozoal population (log_10_ cell/mL)							
0 h pre-feeding	1.33	1.56	1.38	1.25	0.09	0.62	0.50
4 h post-feeding	1.75	1.88	1.69	1.41	0.10	0.58	0.12

1) Contrast: (1) Compare the difference between T1 VS T2, T3, T4, (2) Compare the difference between T3 VS T4 (p<0.05, p<0.01), T1 = control group; T2 = replacement SBM with powdered BSFL 50%; T3 = replacement SBM with pelleted BSFL 25%; T4 = replacement SBM with pelleted BSFL 75%.

SBM, soybean meal; BSFL, black soldier fly larvae; SEM, standard error of the mean.

**Table 5:** Effect of replacement SBM with BSFL on total volatile fatty acids, and volatile fatty acid profile in Thai native cattle

Item	Treatment				SEM	Contrast^1)^	
	
	T1	T2	T3	T4		(^1)^	(2)
Total volatile fatty acid profiles (mmol/L)							
0 h pre-feeding	102.5	98.8	101.2	89.7	7.98	0.53	0.33
4 h post-feeding	98.3	110.9	107.7	110.9	10.9	0.38	0.84
Volatile fatty acid profiles (%)							
Acetic acid							
0 h pre-feeding	62.2	64.9	63.7	62.0	1.04	0.37	0.36
4 h post-feeding	63.9	66.9	66.1	59.9	2.56	0.78	0.40
Propionic acid							
0 h pre-feeding	23.1	20.1	21.8	24.3	1.31	0.60	0.34
4 h post-feeding	21.6	22.8	20.1	26.2	2.54	0.74	0.29
Mean	22.3	21.5	20.9	25.3	1.60	0.92	0.21
Butyric acid							
0 h pre-feeding	14.7	15.0	14.5	13.6	1.11	0.81	0.74
4 h post-feeding	14.5	10.3	13.8	13.9	1.06	0.92	0.77
Acetic/propionic acid ratio							
0 h pre-feeding	2.69	3.22	2.92	2.55	0.19	0.49	0.31
4 h post-feeding	2.96	2.93	3.29	2.28	0.46	0.76	0.31

1) Contrast: (1) Compare the difference between T1 VS T2, T3, T4, (2) Compare the difference between T3 VS T4 (p<0.05, p<0.01), T1 = control group; T2 = replacement SBM with powdered BSFL 50%; T3 = replacement SBM with pelleted BSFL 25%; T4 = replacement SBM with pelleted BSFL 75%.

SBM, soybean meal; BSFL, black soldier fly larvae; SEM, standard error of the mean.
